# Association of Food-Specific Glycemic Load and Distinct Dietary Components with Gestational Diabetes Mellitus Within a Mediterranean Dietary Pattern: A Prospective Cohort Study

**DOI:** 10.3390/nu17111917

**Published:** 2025-06-03

**Authors:** Antigoni Tranidou, Antonios Siargkas, Emmanouela Magriplis, Ioannis Tsakiridis, Panagiota Kripouri, Aikaterini Apostolopoulou, Michail Chourdakis, Themistoklis Dagklis

**Affiliations:** 1 3rd Department of Obstetrics and Gynecology, School of Medicine, Faculty of Health Sciences, Aristotle University of Thessaloniki, 541 24 Thessaloniki, Greece; antigoni.tranidou@gmail.com (A.T.); antonis.siargkas@gmail.com (A.S.); igtsakir@auth.gr (I.T.); p_kripouri@yahoo.com (P.K.); 2 Laboratory of Hygiene, Social & Preventive Medicine and Medical Statistics, School of Medicine, Faculty of Health Sciences, Aristotle University of Thessaloniki, 541 24 Thessaloniki, Greece; katapost@yahoo.gr (A.A.); mhourd@auth.gr (M.C.); 3 Department of Food Science and Human Nutrition, Agricultural University of Athens, Iera Oos 75, 118 55 Athens, Greece; emagriplis@eatsmart.gr

**Keywords:** maternal diet, glycemic index, glycemic load, dietary patterns, factor analysis, gestational diabetes, GDM

## Abstract

**Background/Objectives**: Gestational diabetes mellitus (GDM) is a major pregnancy complication with rising global prevalence. The Mediterranean Diet (MD) has shown metabolic benefits, but total adherence scores may obscure meaningful variation in dietary quality. This study aimed to investigate whether specific dietary patterns, identified within the MD framework, and their glycemic load (GL) are associated with GDM risk. **Methods**: This prospective cohort is part of the BORN2020 longitudinal study on pregnant women in Greece; dietary intake was assessed using a validated food frequency questionnaire (FFQ) at two time points (pre-pregnancy and during pregnancy). MD adherence was categorized by Trichopoulou score tertiles. GL was calculated for food groups using glycemic index (GI) reference values and carbohydrate content. Dietary patterns were identified using factor analysis. Logistic regression models estimated adjusted odds ratios (aORs) for GDM risk, stratified by MD adherence and time period, controlling for maternal, lifestyle, and clinical confounders. **Results**: In total, 797 pregnant women were included. Total MD adherence was not significantly associated with GDM risk. However, both food-specific GLs and dietary patterns with distinct dominant foods were predictive. GL from boiled greens/salads was consistently protective (aOR range: 0.09–0.19, *p* < 0.05). Patterns high in tea, coffee, and herbal infusions before pregnancy were linked to increased GDM risk (aOR = 1.96, 95% CI: 1.31–3.02, *p* = 0.001), as were patterns rich in fresh juice, vegetables, fruits, legumes, and olive oil during pregnancy (aOR = 2.91, 95% CI: 1.50–6.24, *p* = 0.003). A pattern dominated by sugary sweets, cold cuts, animal fats, and refined products was inversely associated with GDM (aOR = 0.34, 95% CI: 0.17–0.64, *p* = 0.001). A pattern characterized by sugar alternatives was associated with higher risk for GDM (aOR = 4.94, 95% CI: 1.48–19.36, *p* = 0.014). These associations were supported by high statistical power (power = 1). **Conclusions**: Within the context of the MD, evaluating both the glycemic impact of specific food groups and identifying risk-associated dietary patterns provides greater insight into GDM risk than overall MD adherence scores alone.

## 1. Introduction

Gestational diabetes mellitus (GDM) has emerged as a significant public health challenge, reflecting global increases in obesity, sedentary lifestyles, and advancing maternal age [1]. It is associated with numerous adverse maternal and fetal outcomes, including hypertensive disorders of pregnancy, fetal macrosomia, shoulder dystocia, and higher rates of cesarean delivery [2]. Beyond immediate pregnancy complications, GDM predisposes both mother and child to long-term metabolic issues, notably an elevated risk of developing type 2 diabetes mellitus and metabolic syndrome in later life [3]. Consequently, timely identification and effective management strategies for GDM have become integral components of contemporary obstetric care.

Dietary carbohydrate intake is central to regulating glucose metabolism, emphasizing the role of both carbohydrate quality and quantity in the development of GDM [4]. While total carbohydrate intake influences glucose levels [5], the quality of consumed carbohydrates, reflected by metrics, such as glycemic index (GI) and glycemic load (GL), is equally important [6]. However, a recent meta-analysis revealed conflicting findings on the association between GI, GL, and GDM prevalence, likely due to variations in dietary patterns and inadequate adjustments for confounders across studies [7]. Relying on GI and GL alone fails to capture the full complexity of an individual’s diet.

Recognizing the limitations of investigating isolated dietary components, research increasingly emphasizes their investigation in the context of holistic dietary patterns [8]. The Mediterranean diet (MD), for instance, has demonstrated protective effects against GDM [9]. Yet, the specific contribution of individual dietary elements within such beneficial patterns, including GI and GL, requires further elucidation. Research has shown that minimal shifts in a broader diet can have significant results. For example, a randomized controlled trial depicted that an MD when supplemented with extra virgin olive oil and nuts, was associated with reduced GDM incidence by approximately 6% [10]. To date, limited research has explored the interplay between GI/GL and other dietary constituents within the framework of the MD, which may mask or amplify their impact on GDM risk. Only one study, to the best of our knowledge, has investigated these associations [11]. This cohort study integrated the GI in the carbohydrate quality index, which was used for the analysis. GI was derived for each food and beverage item using reference tables and adherence to the MD was computed using the nine-item score proposed by Trichopoulou et al. [12]. They concluded that the quality of dietary carbohydrates was not significantly associated with the incidence of GDM in the context of the MD [11]. Other investigations adjusting for broader dietary patterns or energy intake, similarly reported no significant link between GI and GDM, although consumption of GL higher than the 3rd tertile was associated with an increased risk for GDM [13,14].

These findings highlight a critical gap in our understanding and the need to examine individual dietary components of the MD that may modulate glycemic outcomes. This study aims to investigate the association of various distinct dietary patterns, including GI and GL, with the risk of developing GDM among women adhering to an MD. Utilizing multivariable models, we seek to identify which specific dietary components significantly contribute to GDM risk, adjusting for potential confounders, thereby providing more clinically relevant insights into dietary prevention strategies.

## 2. Materials and Methods

### 2.1. Population Characteristics

The present study was conducted within the BORN2020 longitudinal cohort, focusing on the interplay between nutrition, physical activity, and pregnancy outcomes in Northern Greece [15]. Participants were recruited during routine first-trimester ultrasound appointments at the 3^rd^ Department of Obstetrics and Gynecology, School of Medicine, Faculty of Health Sciences, Aristotle University of Thessaloniki. The study included all eligible women enrolled in the BORN2020 cohort between July 2020 and October 2022. A formal a priori sample size calculation was not performed; however, post hoc power analyses were conducted to assess the robustness of significant findings. Women were deemed eligible if they were over 18 years old, the pregnancy did not result in miscarriage or termination of pregnancy and possessed adequate fluency in Greek to ensure precise reporting of dietary habits. Women following specific dietary regimens for health-related reasons were not included, as were those presenting pre-existing type 1 or type 2 diabetes mellitus, chronic hypertension, or autoimmune conditions. In addition, participants diagnosed with GDM before the standard screening window of 24–28 weeks of gestation, typically those at significantly elevated risk, were excluded. The study received approval by the Bioethics Committee of the Aristotle University of Thessaloniki, Greece (protocol code 6.231/Date of approval 29 July 2020).

### 2.2. GDM Diagnosis

GDM diagnosis followed guidelines outlined by the Hellenic Society of Obstetricians and Gynecologists, aligned with criteria established by the HAPO study [16]. Briefly, all participants underwent a 75 g OGTT between 24 and 28 weeks of gestation, with plasma glucose measurements recorded at fasting, 60 min and 120 min. GDM was diagnosed if any of the measured values met or exceeded the cutoffs of 92 mg/dL (fasting), 180 mg/dL (60-min), or 153 mg/dL (120-min). Individuals who had been subject to an earlier OGTT, typically administered to those exhibiting risk factors, were excluded from the study if GDM was diagnosed before 24 weeks, ensuring that all recorded outcomes were based on a uniform diagnostic window.

### 2.3. Food Groups from FFQ

At both time points, trained nutritionists or study staff administered a previously validated semi-quantitative Food Frequency Questionnaire (FFQ) designed for the Greek population [17]. The FFQ recorded consumption frequency, measured in times per day, week, or month, alongside typical portion sizes for a wide array of foods representative of Greek cuisine. Dietary interviews lasted approximately 20 min each, and participants were encouraged to clarify serving sizes and ingredients of composite dishes to mitigate recall bias and reporting inaccuracies. Participants with incomplete or missing FFQ responses were excluded from the analysis. Nutrient calculations were subsequently conducted using Nutrisurvey software (EBISpro, Willstatt, Germany, version 2007). Food items reported in the FFQ were grouped into nutritionally and culturally relevant categories to facilitate factor analysis and GL computation. Groupings were based on macronutrient profile, carbohydrate content, and traditional dietary patterns. For example, non-refined grains included wholegrain bread and pasta, while sugary sweets and sugar beverages comprised desserts with sugar, sugar-sweetened beverages, and packaged juice. Separate groups were created for foods like boiled vegetables (boiled salads), plant-based oils and nuts, and sugar alternatives (e.g., stevia-based products and low-calorie soft drinks).

### 2.4. Dietary Analysis

Analyses were stratified by adherence level to the MD based on the Trichopoulou scoring system (high, medium, low adherence, using tertiles separation) [12]. For each dietary component, we first calculated the cohort median intake (in grams/day or as a ratio) separately for the pre-pregnancy (A) and pregnancy (B) periods. Participants received one point for each “beneficial” component—vegetables, legumes, fruits and nuts, cereals (whole grains), and fish, when their intake was at or above the median, and one point for each “detrimental” component—meat and meat products, and dairy, when their intake was below the median. An additional point was awarded if the ratio of monounsaturated to saturated fats met or exceeded its median. In the pre-pregnancy period only, one point was also assigned for alcohol intake between 5 g and 25 g per day (no alcohol points were given during pregnancy). Thus, total scores ranged from 0 (no adherence) to 9 in period A and to 8 in period B. Participants were then stratified into tertiles within each period [low adherence (0–3 points), medium adherence (4–6 points), and high adherence (7–9 points for pre-pregnancy, 7–8 points for pregnancy)] and all subsequent analyses were conducted within these tertiles to assess effect modification by overall MD quality. GL assessment was chosen over GI alone, since GI does not account for portion size and has limited relevance when evaluating mixed meals or complete dietary patterns. This way, results reflect better the overall dietary glycemic impact, incorporating both carbohydrate quality and quantity. GL for each food group was computed using the formula:

GL = (GI × carbohydrate content per serving [g])/100 × frequency of consumption.

GI values were obtained from international reference tables [18]. Carbohydrate content per serving was derived using NutriSurvey software, based on standard serving sizes as reported in the FFQ. Frequency of intake was extracted directly from FFQ data and converted to daily equivalents. Food groups with negligible carbohydrate content and without established GI values (e.g., meat, eggs, fish, water, plant-based oils) were excluded from GL analyses but retained in dietary pattern modeling.

Food groups with sparse consumption data (e.g., sugar alternatives, legumes, animal fats) produced unstable estimates in logistic regression, and wide confidence intervals were interpreted with caution. These values were retained for completeness but should be viewed as exploratory.

### 2.5. Statistical Analysis

Regarding the population characteristics, for variables following a normal distribution, mean and SD are provided; otherwise, the median and quartiles are reported. The Shapiro–Wilk (<50 samples) or Kolmogorov–Smirnov (>=50 samples) tests were used to assess normality. The *p*-values were obtained using a *t*-test for normally distributed variables, Mann–Whitney test for non-normally distributed variables, and chi-squared test for categorical variables. Fisher’s exact test (n < 5) or chi-squared test (n >= 5) were applied for binary variables, contingent on the sample size.

Statistical analysis was performed using logistic regression models to compute adjusted odds ratios (aORs) and their corresponding 95% confidence intervals (CIs) for the association between dietary variables and the risk of GDM. Dietary exposures included consumption frequency for each food group (portions/day) and GL/day computed for each food group. Factor analysis, employing Varimax rotation, was used to identify prominent dietary patterns from the consumption of food groups. Items with factor loadings ≥0.3 were considered significant contributors to a factor. Food groups with factor loadings ≥0.3 were used to define dietary patterns. All logistic models were adjusted for: total energy intake (kcal/day), maternal age, pre-pregnancy body mass index (BMI), physical activity (walking), thyroid status, parity (nulliparous vs. ≥1), smoking, assisted reproductive technology (ART) use, supplement use, and gestational weight gain. All analyses were conducted using R version 4.2.1.

## 3. Results

**Figure 1:**
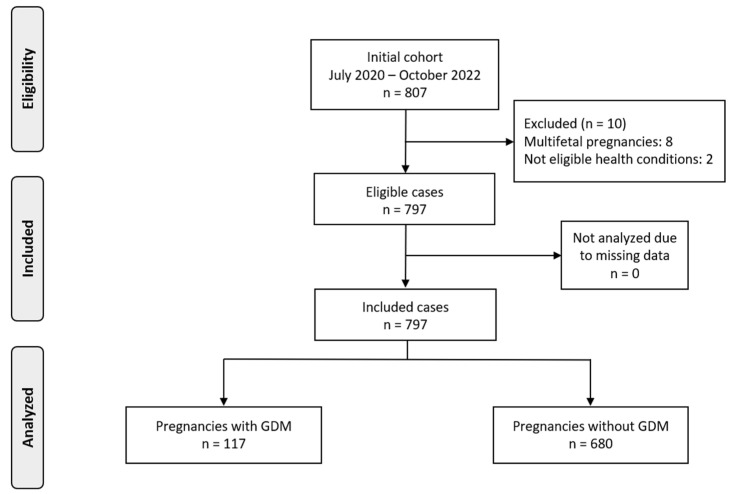
Flowchart of population recruitment.

During the recruitment, we collected data for 807 pregnancies, of which 797 were eligible. From the included population, 117 women (14.7%) were diagnosed with GDM and compared to 680 women without GDM (Figure 1).

Women who developed GDM were significantly older (mean age 34.15 ± 4.48 years vs. 32.1 ± 4.89 years, *p* < 0.0001) and more likely to be of advanced maternal age, with 43.6% over 35 years compared to 27.4% in the non-GDM group (*p* < 0.001). Pre-pregnancy BMI was also significantly higher among women with GDM, with a median of 23.7 kg/m^2^ (interquartile range (IQR): 21.7–28.5) compared to 22.7 kg/m^2^ (IQR: 20.8–26.0) in the non-GDM group (*p* = 0.004). The prevalence of pre-pregnancy obesity was nearly double in the GDM group (21.4% vs. 11.0%, *p* = 0.003). Similar differences were observed for BMI during pregnancy, with obesity present in 25.6% of GDM women versus 11.9% in the non-GDM group (*p* < 0.001). Smoking was more common in the GDM group (17.95% vs. 8.82%, *p* = 0.004), while there were no significant differences in parity, use of assisted reproductive technology (ART), or thyroid disease (all *p* > 0.4) (Table 1).

Table 2 presents MD adherence scores and their distribution across GDM status. No statistically significant differences were observed in total MD scores, with median values of 4 (IQR: 3–6) in the GDM group versus 5 (IQR: 3–6) in the non-GDM group before pregnancy (*p* = 0.58), and both groups reporting a median of 5 during pregnancy (*p* = 0.45). The proportion of women classified into low, medium, and high MD adherence tertiles was similar between groups across both time periods (all *p* > 0.3). However, when MD score components were analyzed individually, stratified by MD adherence level (Supplementary Tables S2–S4), several associations with GDM risk emerged. Among women with high MD adherence, fish consumption was significantly protective against GDM both before pregnancy (aOR = 0.0002, 95% CI: 2.94 × 10^−7^ to 0.11, *p* = 0.013) and during pregnancy (aOR = 0.0008, 95% CI: 1.27 × 10^−6 ^to 0.2, *p* = 0.022). In the medium MD adherence group, consumption of legumes (aOR = 1.57 × 10^4^;, 95% CI: 42.02 to 1.19 × 10^7^;, *p* = 0.002), fruits and nuts (aOR = 1.86, 95% CI: 1.04–3.46, *p* = 0.04), meat (aOR = 18.86, 95% CI: 1.31–286.09, *p* = 0.029), and eggs (aOR = 9.25, 95% CI: 2.07–46.24, *p* = 0.004) during pregnancy was associated with increased GDM risk. For women with low MD adherence, meat consumption before and during pregnancy was also associated with increased GDM risk (aOR = 5.87, 95% CI: 1.67–19.98, *p* = 0.005; and aOR = 4.34, 95% CI: 1.04–17.94, *p* = 0.042, respectively).

A full list of food groups and their constituent FFQ items is provided at the Supplementary Materials tables (Table S1). Median daily intakes of each food group derived from FFQ data are summarized below. Women who developed GDM reported higher intake of non-refined grains both pre-pregnancy (median: 0.286 vs. 0.033 portions/day, *p* = 0.04) and during pregnancy (0.462 vs. 0.143 portions/day, *p* < 0.001). Meat intake was also elevated in the GDM group both pre-pregnancy (0.429 vs. 0.319 portions/day, *p* = 0.018) and during pregnancy (0.429 vs. 0.352 portions/day, *p* = 0.029). Dairy intake was significantly higher during pregnancy among women with GDM (2 vs. 1.1 portions/day, *p* = 0.036), while beverage consumption was substantially higher before pregnancy (median: 2 vs. 1 portions/day, *p* < 0.0001). Egg intake during pregnancy was slightly higher in the GDM group (*p* = 0.039), and ultra-processed food intake during pregnancy was significantly lower (1.35 vs. 1.63 portions/day, *p* = 0.013) (Supplementary Table S5).

Three dietary patterns (Table 3) were extracted through factor analysis for each time period, explaining 15.5% and 12.2% of the total variance in dietary intake pre-pregnancy and during pregnancy, respectively (Tables S6 and S7 respectively). While modest, these values are consistent with previous literature using food frequency questionnaires in free-living populations [19]. Full loading matrices and food group contributions are available in Supplementary Tables S8 and S9.

Table 4 shows which patterns of eating extracted by factor analysis were linked to GDM based on MD adherence level. Only statistically significant results are presented in the manuscript. Table S10 shows the remaining non-statistically significant results.

In the high MD adherence group, the beverage-heavy pattern in the pre-pregnancy period (Factor 3–A) was significantly associated with increased GDM risk (aOR = 1.96, 95% CI: 1.31–3.02, *p* = 0.001). In the medium adherence group, a plant-rich dietary pattern during pregnancy (Factor 1–B), characterized by a high intake of fresh juice, vegetables, fruits, legumes, and nuts, was associated with increased GDM risk (aOR = 2.91, 95% CI: 1.50–6.24, *p* = 0.003). The processed/sweet pattern during pregnancy (Factor 2–B) was inversely associated with GDM (aOR = 0.34, 95% CI: 0.17–0.64, *p* = 0.001). A sweetener-focused pattern (Factor 3–B), dominated by sugar alternatives, was significantly associated with increased GDM risk in both the medium adherence group (aOR = 4.94, 95% CI: 1.48–19.36, *p* = 0.014) and the low adherence group (aOR = 2.16, 95% CI: 1.23–3.85, *p* = 0.008) during pregnancy.

We also examined the association between GL of individual food groups and GDM risk, using both stratified adjusted logistic regression models by MD adherence level and non-stratified models, across periods A and B. Table 5 shows the significant results, while in Tables S11–S14 the total of the results are presented.

Associations between glycemic load (GL) of food groups and GDM risk are shown in Table 5. GL from boiled salads was consistently associated with reduced GDM risk in the high MD adherence group before pregnancy (aOR = 0.09, 95% CI: 0.01–0.73, *p* = 0.032) and during pregnancy (aOR = 0.11, 95% CI: 0.01–0.76, *p* = 0.039). In the medium adherence group, GL from non-refined grains was positively associated with GDM both before pregnancy (aOR = 1.02, 95% CI: 1.01–1.03, *p* < 0.001) and during pregnancy (aOR = 1.01, 95% CI: 1.00–1.02, *p* = 0.005). GL from ultra-processed foods was inversely associated with GDM in this group before pregnancy (aOR = 0.99, 95% CI: 0.99–0.99, *p* = 0.037). In the same group, gL from fruits was also associated with increased GDM (aOR = 1.03, 95% CI: 1–1.07, *p* = 0.022). Similar associations were observed in the low adherence group, with increased risk linked to GL from non-refined grains before pregnancy (aOR = 1.02, 95% CI: 1.01–1.03, *p* < 0.001) and also during pregnancy (aOR= 1.01, 95% CI: 1–1.02, *p* = 0.005), and reduced risk associated with GL from ultra-processed foods (aOR = 0.99, 95% CI: 0.99–0.99, *p* = 0.027). In the non-stratified population, GL from boiled salads remained inversely associated with GDM before pregnancy (aOR = 0.19, 95% CI: 0.05–0.63, *p* = 0.008), while GL from non-refined grains was positively associated with GDM both before and during pregnancy (aOR = 1.01, 95% CI: 1.00–1.02, *p* = 0.005 and 0.002, respectively). GL from ultra-processed foods again showed an inverse association with GDM in both periods (aOR = 0.99, 95% CI: 0.99–0.99, *p* = 0.021 and *p* = 0.037). Supplementary Tables S11–S14 show GL of all food groups across MD adherence levels and the non-stratified population.

## 4. Discussion

This prospective cohort study explored how specific dietary patterns and the GL of individual food groups relate to GDM risk within the broader context of adherence to an MD, including both stratified and non-stratified models. The main findings suggest that total MD adherence, as measured by the Trichopoulou score (grouped into tertiles), was not significantly associated with GDM risk, either before or during pregnancy. Instead, several distinct dietary exposures, both patterns and food specific GL, showed consistent associations across both MD adherence groups and the total population (non-stratified models), highlighting the importance of examining dietary patterns and their GL beyond composite MD scores.

To explore dietary heterogeneity, we conducted a factor analysis to derive dietary patterns based on food group consumption and stratified them by MD adherence level (low, medium, high). Our approach reflects prior studies identifying dietary patterns linked to GDM risk [20,21,22,23]; however, several of our associations diverged from the expected direction, particularly the increased risk seen with a plant-rich pattern and the inverse association with processed patterns. Additional results from supplementary tables, including median intakes, MD adherence food groups and food group specific GL, are also discussed to provide further context.

Dietary patterns derived from factor analysis revealed strong associations with GDM risk, especially in stratified models. In particular, in women with high MD adherence before pregnancy, a beverage-heavy pattern, including tea, herbal infusions, and coffee, was significantly linked to higher GDM risk. During pregnancy, those in the medium adherence group following a plant-rich pattern (fresh juice, fruits, vegetables, legumes, nuts, olives, and olive oil) also faced increased risk. A sugar-alternatives pattern, dominated by non-nutritive sweeteners (diet/light soft drinks, stevia etc.), conferred elevated risk in both medium and low adherence strata during pregnancy. Conversely, a processed/sweetpattern (sugary sweets and beverages, animal fats, cold cuts, ultra-processed foods, refined products) during pregnancy was associated with lower GDM risk among medium-adherence women, likely reflecting reverse causality, as pregnant women tend to reduce these energy-dense items.

Regarding GL analyses, GL from boiled greens/salads, a traditional Greek dish of lightly cooked leafy or non-starchy vegetables, was consistently protective against GDM, especially in high adherence and non-stratified groups across both periods. This protective association was consistent in both periods, suggesting that habitual intake of high-fiber vegetable dishes may contribute to reduced metabolic risk. Although boiled salads as a discrete food group have not been isolated in previous studies, diets rich in vegetables, especially non-starchy types like leafy greens, legumes, and other fibrous vegetables, have been consistently associated with a lower GDM risk [24]. Moreover, in a randomized controlled trial, in women with GDM showing that supplementing the diet with whole berries and leafy vegetables led to significant improvements in postprandial blood glucose, antioxidant status, and inflammatory cytokines, supporting the role of these foods in modulating metabolic and inflammatory pathways relevant to GDM [25]. Boiled greens and salads are typically low in energy density, rich in micronutrients and dietary fiber, and rarely consumed with added sugars or refined starches, characteristics that align with findings that higher intake of polyphenols and flavonoids from plant foods is inversely associated with GDM risk, further supporting their beneficial metabolic profile in pregnancy [26]. 

By contrast, GL from non-refined grains (wholegrain bread, pasta) showed modest but statistically consistent risk increases (aORs 1.01–1.02) in low adherence (Table S13) and non-stratified groups (Table S14), though power was limited (0.058–0.064), suggesting these findings be viewed as hypothesis-generating. Despite the common perception that these foods are metabolically beneficial [27], their high carbohydrate density and large portion sizes may contribute substantially to total dietary GL. This is supported by prior evidence indicating that women in the highest tertiles of total dietary GL during pregnancy had a 43% higher risk of developing GDM [13], and women in the highest GL tertile had a 15–25% higher risk of developing GDM [14]. In our analysis, the higher median intake of non-refined grains in the GDM group aligns with these earlier findings, but future studies with adequate power are needed to clarify this association. Notably, GL from ultra-processed foods, cold cuts, and refined products, a food group category considered metabolically unfavorable, yielded a borderline inverse association with GDM, observed across low adherence levels (Table S13) and in the total sample (Table S14). However, this finding rests on very low statistical power (power ≈ 0.05) and minimal effect size. It may therefore reflect reverse causality or behavioral compensation, whereby women who perceive themselves at higher metabolic risk reduce their intake of these energy-dense, processed foods following medical or dietary advice, rather than indicating any true protective effect.

While the MD is widely acknowledged to benefit metabolic health, its relationship with GDM remains complex, particularly because composite adherence scores may obscure important variations in dietary quality and overlook the metabolic effects of specific dietary behaviors [21,28]. Some studies have reported a protective effect of MD adherence [29,30], while others, particularly those not accounting for within-score dietary quality, emphasize the importance of dissecting composite scores into their component dietary behaviors [22,31]. Our findings underscore the complexity of diet-disease relationships, as some dietary patterns, like the plant-rich and processed patterns, diverged from expected directions, highlighting the need for further research, considering both dietary quality and context is crucial when assessing metabolic risk in pregnancy.

Focusing on the high MD adherence tertile, a beverage-heavy pattern, dominated by tea, herbal infusions and coffee, was significantly associated with increased GDM risk before pregnancy. This was supported by higher median beverage intake before pregnancy (Table S5) among women who developed GDM, aligning with a recent Greek cohort study by Tsarna et al. [31]. While their analysis focused on tea consumption during pregnancy, they noted measurement limitations, such as lack of information on sweetening and retrospective recall bias. Our study assessed both non-nutritive sweeteners (via sugar alternatives food group: low calorie soft drinks, alternative sweeteners) and nutritive sweeteners (via the “added teaspoons of sugar or honey” item within the sugary sweets and sugar beverages group which also includes sugar-sweetened desserts, regular soft drinks, and packaged juice; (Table S1). Median intake of sugar alternatives did not differ significantly between GDM and non-GDM groups. The observed association may reflect early metabolic disturbances or compensatory behaviors among women at elevated risk as has been previously reported [32]. While some herbs have shown potential to improve glucose metabolism [33], there is currently no direct evidence linking herbal infusion consumption before pregnancy to a reduced or increased risk of GDM. More targeted research is needed in this area. Although our findings highlighted a pre-pregnancy association, existing evidence suggests that moderate tea and coffee intake during pregnancy is not linked to increased GDM risk when consumed in moderation [34], highlighting the potential influence of timing, quantity, and dietary context.

In the medium MD adherence group, a plant-rich pattern, characterized by fresh juice, vegetables, fruits, legumes, nuts, olives, and olive oil, was associated with increased GDM risk during pregnancy. This may be attributable to the higher GL of certain components, such as fruit juices and starchy vegetables. Prior work by Li et al. linked higher first-trimester starchy vegetable intake, including potatoes, with elevated GDM risk [35]. In our stratified models, GL from legumes and fruits, including dried fruits which are particularly energy- and sugar-dense (Table S12), were associated with increased GDM risk, athough some estimate for legumes showed considerable imprecision. Notably, the association between fruit GL and GDM, although statistically significant, was accompanied by low post hoc power (0.062), suggesting that this finding should be interpreted cautiously. While low-GI fruits are associated with decreased risk for GDM [36], high fruit intake in the second trimester of pregnancy (increasing overall fruit GL) may increase GDM risk nearly fivefold [37]. On the other hand, consumption of fruits with low or moderate GI (like apples and oranges) may be linked to reduced GDM risk, while high-GI fruits and fruit juice are related to an increased risk [38]. 

Additionally, in the same medium-adherence group, a sweet/processed pattern that included sugary snacks, animal fats, cold cuts, and ultra-processed products was inversely associated with GDM. In general, this type of diet has been associated with increased GDM risk [23]. One possible explanation of this is reverse causality: women at perceived metabolic risk may have consciously restricted intake of these items, leading to lower reported consumption of ultra-processed foods among GDM cases compared to the non-GDM individuals.

Building on the factor analysis framework, another dietary pattern, dominated by sugar alternatives, was consistently associated with increased GDM risk in both medium and low MD adherence groups, specifically during pregnancy. The observed aORs were large (e.g., 4.94 in the medium group), but accompanied by wide confidence intervals. This could reflect either a true adverse metabolic effect of artificial sweeteners, as suggested by emerging literature linking them to altered insulin sensitivity and gut microbiota [39], or reverse causality, where higher-risk women turn to sugar alternatives as a perceived healthier option. Prior studies also support this possibility: one cohort reported a 166% higher GDM risk among high consumers of artificial sweeteners during pregnancy, even after stratifying by BMI [40], while another study similarly linked non-nutritive sweetener intake to elevated GDM risk [41]. Interestingly, this pattern did not emerge among high MD adherence women, raising the possibility that dietary context may modify the impact of specific food components.

Some associations identified with the MD stratification produced wide confidence intervals, suggesting the need for caution. For instance, fish intake in high MD adherence women (Table S2) was associated with a strong protective effect (aOR = 0.0002), but the confidence interval was extremely wide, reflecting limited consumption. Similarly, large but imprecise effect sizes were seen for legumes and sugar alternatives in the medium adherence group. These findings should be interpreted as exploratory. Nonetheless, several results are supported by prior literature. Fish intake has been widely associated with improved glucose metabolism and reduced GDM risk due to its omega-3 content [42]. Similarly, higher meat (Tables S3 and S4) and egg (Table S3) intake was associated with elevated GDM risk in the medium and lower MD adherence groups, reflecting prior evidence linking red and processed meats to impaired glucose metabolism and dietary heme-iron intake [43].

These findings reinforce the idea that diet should be considered as a whole, rather than focusing on isolated food items [44]. Nutrients are consumed within broader dietary patterns, where interactions among components can influence overall metabolic effects [45]. While the MD is widely promoted for metabolic health, our results suggest that its benefits may depend on the quality and context of individual dietary components. For example, the apparent protective effect of ultra-processed patterns in some groups may reflect behavioral compensation, while traditional low-GL components, like boiled salads, appear consistently beneficial. These observations align with previous research linking high intake of red meats, sugar substitutes, and ultra-processed products to increased GDM or type 2 diabetes risk, even after controlling for lifestyle and socioeconomic factors [21,31,46].

Key strengths of this study include its prospective design, the use of validated FFQ data collected at two time points, pre-pregnancy and during pregnancy, and comprehensive adjustment for a broad range of maternal, lifestyle, and clinical confounders. Stratification by MD adherence and the application of factor analysis allowed for a more nuanced exploration of dietary quality within adherence levels, which is often overlooked in studies relying solely on composite diet scores. Unlike most prior studies that assessed total dietary GL, our analysis estimated glycemic load separately for specific food groups, enabling the identification of dietary components most strongly associated with metabolic risk. This food-specific approach provides more granular insight into the glycemic profile of the MD and may help explain some of the counterintuitive associations observed.

This work should also be interpreted in light of several limitations. First, dietary intake was self-reported using a semi-quantitative FFQ, making it vulnerable to recall and social desirability bias, despite the tool’s prior validation. Second, glycemic index values were derived from international reference tables rather than direct testing of local products, which may have introduced measurement error, particularly for composite dishes. Third, the cohort was recruited exclusively in Northern Greece, where consumption of “boiled greens/salads” (lightly boiled wild or cultivated greens with olive oil and lemon) is common. As this dish is uncommon, or prepared differently, in other cultural settings, the protective association we observed may not generalize. Replication in other populations or in experimental designs will be needed to establish external validity. Fourth, some food groups (e.g., fish, legumes) resulted in wide confidence intervals and reduced power to detect modest associations. Finally, although extensive adjustments were made for known confounders, residual and unmeasured confounding, such as genetic variation, gut microbiota, or insulin sensitivity, cannot be excluded.

In summary, our findings suggest that food-specific glycemic load, portion size, and dietary context may be more informative than total MD adherence for predicting GDM risk. By stratifying by MD adherence and capturing intake at both the pre-pregnancy and pregnancy stages, our study offers insight into how dietary behaviors may influence metabolic risk across critical reproductive time points.

While this study highlights important associations between food-specific glycemic load, dietary patterns, and GDM risk, further research is warranted to strengthen these findings. Future studies should aim to replicate these results in more diverse populations, especially outside the Mediterranean region, to assess the generalizability of culturally specific dietary practices such as boiled greens. Longitudinal designs with repeated dietary assessments and postnatal follow-up could clarify causal pathways and long-term metabolic consequences. Additionally, intervention studies targeting high-GL food groups or emphasizing protective components like boiled salads could help determine whether modifying specific dietary behaviors reduces GDM incidence. Finally, mechanistic studies exploring how food preparation, glycemic responses, and gut microbiota interact in pregnancy may offer deeper insights into the biological basis of these associations.

## 5. Conclusions

This study shows that not all components of the MD confer equal metabolic benefit. While total MD adherence was not associated with GDM risk, specific foods, particularly those with high GL, were linked to increased risk, even when traditionally considered healthy. GL from boiled greens/salads was consistently protective, while sugar alternatives increased risk. These findings emphasize the need to move beyond broad dietary scores and focus on the quality, context, and timing of specific dietary exposures in GDM prevention.

## Figures and Tables

**Table 1:** Baseline characteristics of women with and without GDM.

Maternal Characteristics	GDM (N = 117)	Non-GDM (N = 680)	*p*-Value
Maternal Age (years)	34.15 (±4.48)	32.1 (±4.89)	*p* < 0.0001 *
Maternal Age > 35 (%)	51 (43.59%)	186 (27.35%)	*p* < 0.001 *
Pre-pregnancy BMI (kg/m^2^)	23.7 (21.7, 28.5)	22.7 (20.8, 26.02)	0.004 *
Pre-pregnancy BMI < 18.5 (%)	2 (1.71%)	28 (4.12%)	0.32
Pre-pregnancy BMI 18.5–24.9 (%)	69 (58.97%)	438 (64.41%)	0.31
Pre-pregnancy BMI 25–29.9 (%)	46 (39.32%)	214 (31.47%)	0.12
Pre-pregnancy BMI ≥ 30 (%)	25 (21.37%)	75 (11.03%)	0.003 *
BMI < 18.5 during pregnancy	1 (0.855%)	20 (2.94%)	0.32
BMI 18.5–24.9 during pregnancy	66 (56.41%)	422 (62.06%)	0.29
BMI 25–29.9 during pregnancy	20 (17.09%)	157 (23.09%)	0.19
BMI ≥ 30 during pregnancy	30 (25.64%)	81 (11.91%)	*p* < 0.001 *
Smoking (%)	21 (17.95%)	60 (8.82%)	0.004 *
Parity			
0	60 (51.28%)	347 (51.03%)	1
1	44 (37.61%)	253 (37.21%)	1
2	12 (10.26%)	69 (10.15%)	1
3	1 (0.855%)	9 (1.32%)	1
4	0 (0%)	2 (0.294%)	-
ART (%)	11 (9.4%)	48 (7.06%)	0.48
Thyroid Disease (%)	13 (11.11%)	93 (13.68%)	0.54

BMI: body mass index; ART: assisted reproductive technology; thyroid disease: hypothyroidism, Hashimoto’s disease; hyperthyroidism; Values are presented as mean ± SD for normally distributed variables and median (IQR) for skewed variables. Differences tested using *t*-tests (normal), Mann–Whitney U tests (non-normal), and chi-squared or Fisher’s exact tests (categorical, based on sample size). * Denotes statistical significance.

**Table 2:** Adherence to Mediterranean Diet score by Trichopoulou and tertiles by GDM status.

Time Period	Adherence to MD	GDM (N = 117)	Non-GDM (N = 680)	*p*-Value
Pre-pregnancy (A)	Total MD Score (median, IQR)	4 (3, 6)	5 (3, 6)	0.58
	Low adherence (%)	N = 61 (52.14%)	N = 332 (48.82%)	0.57
	Medium adherence (%)	N = 19 (16.24%)	N = 141 (20.74%)	0.32
	High adherence (%)	N = 37 (31.62%)	N = 207 (30.44%)	0.88
During pregnancy (B)	Total MD Score (median, IQR)	5 (4, 6)	5 (3, 6)	0.45
	Low adherence (%)	N = 50 (42.74%)	N = 317 (46.62%)	0.5
	Medium adherence (%)	N = 30 (25.64%)	N = 159 (23.38%)	0.68
	High adherence (%)	N = 37 (31.62%)	N = 204 (30%)	0.81

MD: Mediterranean Diet; MD adherence scores categorized by tertiles (low, medium, high) following the Trichopoulou scoring system. Group differences tested using chi-squared tests for categorical adherence.

**Table 3:** Factor analysis results and dietary pattern types at pre-pregnancy (period A) and during pregnancy (period B).

Time Period	Factor	Dominant Food Groups	Pattern Type	Variance Explained (%)
A	Factor 1	Non-refined products and grains, fruits, boiled salad, legumes, nuts and olives and oil	Whole plant-based	6.79%
	Factor 2	Sugary sweets and sugar beverages, vegetables, nuts and olives and oil, animal fats, ultra processed foods and cold cuts and refined	Processed/sweet	5.24%
	Factor 3	Beverages	Beverage-heavy	3.47%
B	Factor 1	Fresh juice, vegetables, fruits, legumes, nuts and olives and oil	Plant-rich with juice	5.12%
	Factor 2	Sugary sweets and sugar beverages, animal fats, ultra processed foods and cold cuts and refined	Western/processed	4.82%
	Factor 3	Sugar alternatives	Sweetener-focused	2.28%

Dietary patterns derived via factor analysis with Varimax rotation, based on FFQ food groups. Factors with loadings ≥0.3 are shown. Total variance explained: 15.5% (pre-pregnancy, period A) and 12.2% (during pregnancy, period B). Pattern names reflect dominant food groups per time period; similar but non-identical loadings led to distinct labels (e.g., processed/sweet vs. western/processed).

**Table 4:** Significant associations between extracted dietary patterns from factor analysis and GDM risk by Mediterranean Diet adherence for periods A and B.

MD Adherence	Time Period	Factor	Dominant Foods	Association	aOR (95% CI)	*p*-Value	Power (aOR)
High	Pre-pregnancy (A)	3–A	Beverages	↑ Risk	1.96 (1.31–3.02)	0.001 *	1
Medium	During pregnancy (B)	1–B	Fresh juice, vegetables, fruits, legumes, nuts and olives and oil	↑ Risk	2.91 (1.50–6.24)	0.003 *	1
Medium	During pregnancy (B)	2–B	Sugary sweets & sugar beverages, animal fats, ultra processed foods & cold cuts & refined	↓ Risk	0.34 (0.17–0.64)	0.001 *	1
Medium	During pregnancy (B)	3–B	Sugar alternatives	↑ Risk	4.94 (1.48–19.36)	0.014 *	1
Low	During pregnancy (B)	3–B	Sugar alternatives	↑ Risk	2.16 (1.23–3.85)	0.008 *	1

MD: Mediterranean Diet; GDM: Gestational Diabetes Mellitus; Adjusted odds ratios (aORs) estimated using logistic regression, stratified by MD adherence level. Models adjusted for maternal age, pre-pregnancy BMI, gestational weight gain, physical activity, smoking, ART use, thyroid status, parity, supplement use, and total energy intake. * Denotes statistical significance.

**Table 5:** Significant glycemic load (GL) associations with GDM risk, across Mediterranean Diet adherence level and the non-stratified population, by time period (A pre-pregnancy and B during pregnancy).

Time Period	MD Adherence	Food Group	aOR (95% CI)	*p*-Value	Power (aOR)
A	High	Boiled salad	0.09 (0.01–0.73)	0.032 *	1
B	High	Boiled salad	0.11 (0.01–0.76)	0.039 *	1
A	Medium	Ultra processed and Cold cuts and refined	0.99 (0.99–0.99)	0.037 *	0.05
B	Medium	Fruits	1.03 (1, 1.07)	0.022 *	0.062
A	Low	Non-refined products and grains	1.02 (1.01–1.03)	<0.001 ***	0.064
B	Low	Non-refined products and grains	1.01 (1, 1.02)	0.005 **	0.057
A	Low	Ultra processed and Cold cuts and refined	0.99 (0.99–0.99)	0.027 *	0.05
A	Non-stratified	Boiled salad	0.19 (0.05–0.63)	0.008 **	1
A	Non-stratified	Non-refined products and grains	1.01 (1.00–1.02)	0.005 **	0.059
A	Non-stratified	Ultra processed and Cold cuts and refined	0.99 (0.99–0.99)	0.021 *	0.05
B	Non-stratified	Non-refined products and grains	1.01 (1.00–1.02)	0.002 **	0.058
B	Non-stratified	Ultra processed and Cold cuts and refined	0.99 (0.99–0.99)	0.037 *	0.05

Glycemic load (GL) values computed as: (GI × carbohydrate content per serving/100) × frequency. MD: Mediterranean Diet; GDM: Gestational Diabetes Mellitus; Adjusted logistic regression models stratified by MD adherence. Confounders: age, BMI, GWG, energy intake, lifestyle, and clinical variables as above. * Denotes statistical significance: * *p* < 0.05; ** *p* < 0.01; *** *p* < 0.001.

## Data Availability

Data are not publicly available due to privacy restrictions.

## Supplementary Materials

The following supporting information can be downloaded at: https://www.mdpi.com/article/10.3390/nu17111917/s1, Table S1: FFQ items and corresponding food groups.; Table S2: High adherence to MD and food components of the MD score, in relation to GDM risk, for periods A and B.; Table S3: Medium adherence to MD and food components of the MD score, in relation to GDM risk, for periods A and B.; Table S4: Low adherence to MD and food components of the MD score, in relation to GDM risk, for periods A and B.; Table S5: Median consumption food groups non stratified. Table S6: Variance explained by factor for Period A.; Table S7: Variance explained by factors for Period B.; Table S8: Loadings period A.; Table S9: Loadings period B.; Table S10: Factors and adherence to MD. Table S11: GL associations with GDM in the high MD adherence group.; Table S12: GL associations with GDM in the medium MD adherence group.; Table S13: GL associations with GDM in the low adherence group.; Table S14: GL associations with GDM non-stratified (total population).
