# Supplementary material for: Association of Food-Specific Glycemic Load and Distinct Dietary Components with Gestational Diabetes Mellitus Within a Mediterranean Dietary Pattern: A Prospective Cohort Study
Source: Nutrients. 2025 Jun 3;17(11):1917. doi: 10.3390/nu17111917 (PMC12157814; doi:10.3390/nu17111917)
Supplement: Supplementary file 1 [file nutrients-17-01917-s001.zip › nutrients-3679061-supplementary.pdf]

Supplementary material

Table S1. FFQ items and corresponding food groups.

**Food groups**	**FFQ items**
**Sugary sweets & Sugar beverages**	- Sugar-sweetened desserts (e.g., cookies, chocolate)
	- Added teaspoons of sugar or honey
	- Regular soft drinks (1 can = 330 ml)
	- Packaged juice (0.5 cup = 125 ml)
**Non-Refined products and grains**	- Whole wheat bread (1 toast slice)
	- Whole grain pasta (1 yogurt container)
**Processed foods**	- Breakfast cereals (e.g., cornflakes)
**Meat**	- Red meat (beef, pork, lamb, goat – palm-sized portion)
	- White meat (chicken, turkey, rabbit – palm-sized)
**Beverages**	- Tea (0.5 cup = 125 ml)
	- Herbal infusions (e.g., chamomile) (0.5 cup)
	- Coffee with caffeine (0.5 cup)
	- Decaf coffee (0.5 cup)
**Fresh Juice**	- Freshly squeezed juice (0.5 cup = 125 ml)
**Alcohol**	- Alcoholic beverages
**Vegetables**	- Potatoes (1 yogurt container)
	- Stewed vegetable dishes (e.g., green beans, peas, ratatouille – 1.5 yogurt containers)
**Fruits**	- Fresh fruits (1 medium or 2 small)
	- Dried fruits (3 pieces for large types)
**Fresh salad**	- Fresh salads (1 yogurt container)
**Boiled salad**	- Boiled greens/salads (0.5 yogurt container)
**Eggs**	- Eggs (1 piece)
**Legumes**	- Legumes (e.g., chickpeas, lentils – 1 yogurt container) (Excludes peas and green beans)
**Nuts, olives, and oils**	- Olive oil (1 tbsp)
	- Olives (5 pieces)
	- Vegetable oils (e.g., corn oil – 1 tbsp)
	- Nuts (1 handful)
**Fish and shellfish**	- Fish and seafood (small fish: 4–5 pieces)
**Plant-based dairy**	- Plant-based dairy alternatives
**Dairy**	- Full-fat dairy (e.g., 250 ml milk or 2 cheese slices)
	- Semi-skimmed dairy (same portion)
**Animal fats**	- Butter (1 tsp)
	- Full-fat cream
	- Low-fat cream
**Plant fats**	- Margarine (1 tsp)
**Sugar alternatives**	- Low-calorie soft drinks (e.g., diet/light)

	- Desserts with alternative sweeteners (e.g., stevia)
**Water**	- Water (1 cup = 250 ml)
**Ultra-processed foods & Cold cuts & Refined**	- Fast food
	- Homemade or phyllo pies (1 piece 6x6 cm)
	- Puff pastries (1 piece 6x6 cm)
	- Salty snacks (e.g., chips)
	- Cold cuts (e.g., ham, turkey – 1 slice)
	- White bread (1 toast slice)
	- Orzo (1 yogurt container)
	- Rice (0.5 yogurt container)
	- White pasta (1 yogurt container)

Adherence to Mediterranean Diet

Table S2. High adherence to MD and food components of the MD score, in relation to GDM risk, for periods A and B.

**Food items by Trichopoulou**	p-value (aOR)	aOR (95% CI)
**Before pregnancy**		
Trihopoulou_Vegetables - A	0.81	0.9 (0.39,1.99)
Trihopoulou _Legumes - A	0.63	0.24 (0,61.15)
Trihopoulou_Fruits and Nuts - A	0.81	0.95 (0.66,1.35)
Trihopoulou_cereals - A	0.86	1.09 (0.36,2.95)
Trihopoulou_fish - A	0.013*	0.0002 ($2.94*10^{-7}$,0.11)
Trihopoulou_ratio - A	0.57	0.8 (0.37,1.68)
Trihopoulou_meat - A	0.42	2.72 (0.21,28.97)
Trihopoulou_dairy products - A	0.89	0.97 (0.59,1.32)
Trihopoulou_Alcohol - A	0.27	2.27 (0.42,9.53)
Trihopoulou_eggs - A	0.54	0.69 (0.19,2.11)
Trihopoulou_sugar and sweets - A	0.057	2.64 (0.97,7.34)
Trihopoulou_nonalcoholic beverages - A	0.99	$8.18*10^{-45}$ (-,inf)
**During pregnancy**		
Trihopoulou_Vegetables - B	0.45	0.74 (0.34,1.55)
Trihopoulou_Legumes - B	0.72	0.41 (0,47.68)
Trihopoulou_Fruits and Nuts - B	0.28	1.2 (0.85,1.7)
Trihopoulou_cereals - B	0.55	1.22 (0.64,2.72)
Trihopoulou_fish - B	0.022*	0.0008 ($1.27*10^{-6}$,0.2)
Trihopoulou_ratio - B	0.45	0.76 (0.36,1.51)
Trihopoulou_meat - B	0.74	1.46 (0.13,13.65)
Trihopoulou_dairy products - B	0.31	1.3 (0.77,2.16)
Trihopoulou_Alcohol - B	0.75	6.62 ($2.76*10^{-6}$,$1.88*10^{5}$)

Trihopoulou_eggs - B	0.27	1.86 (0.6,5.62)
Trihopoulou_sugar and sweets - B	0.32	1.55 (0.63,3.72)
Trihopoulou_nonalcoholic beverages - B	0.99	$2.18*10^{-51}$ (-,inf)

Table S3. Medium adherence to MD and food components of the MD score, in relation to GDM risk, for periods A and B.

**Food items by Trichopoulou**	p-value (aOR)	aOR (95% CI)
**Before pregnancy**		
Trihopoulou_Vegetables - A	0.47	0.63 (0.17,2.07)
Trihopoulou_Legumes - A	0.76	2.68 (0,1199.31)
Trihopoulou_Fruits and Nuts - A	0.2	1.48 (0.8,2.75)
Trihopoulou_cereals - A	0.68	1.27 (0.35,3.89)
Trihopoulou_fish - A	0.15	106.34 (0.11,$5.56*10^{4}$)
Trihopoulou_ratio - A	0.89	1.07 (0.35,2.97)
Trihopoulou_meat - A	0.44	2.89 (0.15,40.39)
Trihopoulou_dairy products - A	0.12	0.55 (0.25,1.06)
Trihopoulou_Alcohol - A	0.2	5.67 (0.24,78.67)
Trihopoulou_eggs - A	0.33	2.24 (0.42,11.63)
Trihopoulou_sugar and sweets - A	0.87	1.08 (0.38,2.7)
Trihopoulou_nonalcoholic beverages - A	-	- (-,-)
**During pregnancy**		
Trihopoulou_Vegetables - B	0.25	1.69 (0.68,4.27)
Trihopoulou_Legumes - B	0.002**	$1.57*10^{4}$ (42.02,$1.19*10^{7}$)
Trihopoulou_Fruits and Nuts - B	0.04*	1.86 (1.04,3.46)
Trihopoulou_cereals - B	0.93	0.94 (0.22,2.91)
Trihopoulou_fish - B	0.45	9.17 (0.01,2331.63)
Trihopoulou_ratio - B	0.39	1.55 (0.56,4.21)
Trihopoulou_meat - B	0.029*	18.86 (1.31,286.09)
Trihopoulou_dairy products - B	0.83	0.94 (0.48,1.52)
Trihopoulou_Alcohol - B	0.56	0 ($5.47*10^{-24}$,$2.17*10^{4}$)
Trihopoulou_eggs - B	0.004**	9.25 (2.07,46.24)
Trihopoulou_sugar and sweets - B	0.23	0.43 (0.09,1.43)
Trihopoulou_nonalcoholic beverages - B	-	- (-,-)

Table S4. Low adherence to MD and food components of the MD score, in relation to GDM risk, for periods A and B.

**Food items by Trichopoulou**	p-value (aOR)	aOR (95% CI)
**Before pregnancy**		
Trihopoulou_Vegetables - A	0.52	0.83 (0.45,1.41)
Trihopoulou_Legumes - A	0.44	4.68 (0.08,225.18)
Trihopoulou_Fruits and Nuts - A	0.97	1 (0.68,1.41)
Trihopoulou_cereals - A	0.61	1.27 (0.47,3.12)
Trihopoulou_fish - A	0.43	0.21 (0,6.63)
Trihopoulou_ratio - A	0.33	1.3 (0.74,2.23)
Trihopoulou_meat - A`	0.005**	5.87 (1.67,19.98)
Trihopoulou_dairy products - A	0.58	1.09 (0.78,1.49)
Trihopoulou_Alcohol - A	0.82	1.19 (0.22,4.61)
Trihopoulou_eggs - A	0.18	1.67 (0.78,3.58)
Trihopoulou_sugar and sweets - A	0.48	0.8 (0.42,1.44)
Trihopoulou_nonalcoholic beverages - A	0.99	$1.64*10^{-91}$ (-,inf)
**During pregnancy**		
Trihopoulou_Vegetables - B	0.44	1.23 (0.72,2.05)
Trihopoulou_Legumes - B	0.47	2.79 (0.15,54.78)
Trihopoulou_Fruits and Nuts - B	0.73	1.06 (0.74,1.46)
Trihopoulou_cereals - B	0.17	0.54 (0.2,1.22)
Trihopoulou_fish - B	0.85	0.68 (0.01,31.24)
Trihopoulou_ratio - B	0.4	1.3 (0.69,2.41)
Trihopoulou_meat - B	0.042*	4.34 (1.04,17.94)
Trihopoulou_dairy products - B	0.39	1.17 (0.81,1.69)
Trihopoulou_Alcohol - B	0.22	20.08 (0.17,5488.72)
Trihopoulou_eggs - B	0.8	1.07 (0.55,1.83)
Trihopoulou_sugar and sweets - B	0.5	0.79 (0.38,1.51)
Trihopoulou_nonalcoholic beverages - B	1	$5.92*10^{-25}$ (-,inf)

Table S5. Median consumption food groups non stratified.

	**GDM (N=117)**	**Non-GDM (N=680)**	**p-value**
**Sugary sweets & Sugar beverages - A**	0.714 (0.286,1.14)	0.714 (0.286,1.07)	0.61
**Non-Refined products and grains - A**	0.286 (0,1)	0.033 (0,0.775)	0.04*
**Processed foods - A**	0.286 (0,0.571)	0.286 (0,0.429)	0.97
**Meat group - A**	0.429 (0.286,0.571)	0.319 (0.286,0.429)	0.018*
**Beverages - A**	2 (1,2)	1 (0.571,2)	p<0.0001**
**Fresh Juice - A**	0 (0,0.429)	0.067 (0,0.429)	0.61
**Alcohol - A**	0.033 (0,0.286)	0 (0,0.143)	0.073
**Vegetables - A**	0.319 (0.286,0.486)	0.352 (0.286,0.429)	0.71

**Fruits - A**	1 (0.286,1.43)	1 (0.429,1.32)	0.86
**Fresh salad - A**	0.714 (0.429,1)	0.857 (0.429,1)	0.81
**Boiled salad - A**	0.067 (0,0.429)	0.143 (0,0.429)	0.079
**Eggs - A**	0.286 (0.067,0.429)	0.286 (0.067,0.429)	0.28
**Legumes - A**	0.143 (0.143,0.143)	0.143 (0.143,0.143)	0.96
**Nuts and olives and oil - A**	2.29 (1.71,3.17)	2.21 (1.43,3.14)	0.14
**Fish and shellfish - A**	0.143 (0.067,0.143)	0.143 (0.067,0.143)	0.27
**Plant-based dairy - A**	0 (0,0)	0 (0,0)	0.9
**Dairy - A**	1.29 (1,2)	1 (1,2)	0.26
**Animal Fats - A**	0.067 (0,0.233)	0.067 (0,0.286)	0.91
**Plant Fats - A**	0 (0,0)	0 (0,0)	0.58
**Sugar alternatives - A**	0 (0,0.033)	0 (0,0)	0.14
**Water - A**	6 (4,8)	6 (4,8)	0.2
**Ultra processed foods & ColdCut & Refined - A**	2.06 (±1.42)	1.92 (±1.26)	0.32
**Sugary sweets & Sugar beverages - B**	0.429 (0.143,1)	0.571 (0.286,1)	0.019*
**Non-Refined products and grains - B**	0.462 (0,1.14)	0.143 (0,1)	p<0.001***
**Processed foods - B**	0.286 (0,0.429)	0.286 (0,0.429)	0.23
**Meat group - B**	0.429 (0.286,0.571)	0.352 (0.286,0.429)	0.029*
**Beverages - B**	1 (0.286,1.13)	1 (0.092,1)	0.15
**Fresh Juice - B**	0.067 (0,0.429)	0.143 (0,0.429)	0.76
**Alcohol - B**	0 (0,0)	0 (0,0)	0.058
**Vegetables - B**	0.352 (0.286,0.429)	0.386 (0.286,0.455)	0.86
**Fruits - B**	1 (0.714,2)	1 (0.571,2)	0.35
**Fresh salad - B**	1 (0.571,1)	1 (0.429,1)	0.48
**Boiled salad - B**	0.133 (0,0.429)	0.143 (0,0.429)	0.93
**Eggs - B**	0.286 (0.1,0.429)	0.2 (0.067,0.429)	0.039*
**Legumes - B**	0.143 (0.143,0.143)	0.143 (0.1,0.143)	0.082
**Nuts and olives and oil - B**	2.43 (1.71,3.43)	2.29 (1.43,3.29)	0.11
**Fish and shellfish - B**	0.143 (0.067,0.143)	0.143 (0.067,0.143)	0.48
**Plant-based dairy - B**	0 (0,0)	0 (0,0)	0.29
**Dairy - B**	2 (1,2)	1.1 (1,2)	0.036*
**Animal Fats - B**	0.067 (0,0.176)	0.067 (0,0.176)	1
**Plant Fats - B**	0 (0,0)	0 (0,0)	0.59
**Sugar alternatives - B**	0 (0,0)	0 (0,0)	0.15
**Water - B**	7.14 (5,8)	6 (4,8)	0.23
**Ultra processed foods & ColdCut & Refined - B**	1.35 (0.714,2.29)	1.63 (0.957,2.66)	0.013*

*Food groups derived from FFQ used in the study.

Factor analysis

Table S6. Variance explained by factor for Period A.

Factor	Variance Explained (%)
Factor 1	6.793559
Factor 2	5.235682
Factor 3	3.474285
Total	15.50353

Table S7. Variance explained by factors for Period B.

Factor	Variance Explained (%)
Factor 1	5.122832
Factor 2	4.821763
Factor 3	2.280068
Total	12.22466

Table S8. Loadings period A.

	Factor1	Factor2	Factor3	H^2 (Communality)
**Sugary sweets & Sugar beverages - A**	-0.19003	0.443925	0.079529	0.239504
**Non-Refined products and grains - A**	0.339873	-0.15567	0.109125	0.151655
**Processed foods - A**	0.204205	-0.05805	-0.0163	0.045335
**Meat group - A**	-0.10912	0.26134	0.031536	0.081201
**Beverages - A**	-0.01378	0.191741	0.731749	0.572412
**Fresh Juice - A**	0.299275	0.026045	-0.18192	0.123338
**Alcohol - A**	-0.01838	-0.04368	0.254128	0.066827
**Vegetables - A**	0.228739	0.307287	-0.14281	0.167143
**Fruits - A**	0.562406	0.003066	-0.00285	0.316318
**Fresh salad - A**	0.283115	-0.15562	0.21446	0.150364
**Boiled salad - A**	0.373824	0.044047	0.07049	0.146654
**Eggs - A**	0.267789	0.02125	0.046833	0.074356
**Legumes - A**	0.381927	0.024464	-0.10694	0.157904
**Nuts and olives and oil - A**	0.422158	0.376813	-0.12466	0.335746
**Fish and shellfish - A**	0.218785	-0.08412	-0.01125	0.05507
**Plant-based dairy - A**	0.209462	-0.23128	-0.01349	0.097547
**Dairy - A**	0.154028	0.203934	0.002159	0.065318
**Animal Fats - A**	-0.00321	0.30485	0.027982	0.093727
**Plant Fats - A**	0.021459	0.152382	-0.01121	0.023807
**Sugar alternatives - A**	0.008905	0.005066	0.067307	0.004635

**Water - A**	0.131225	-0.0843	0.064129	0.028439
**Ultra processed foods & ColdCut & Refined - A**	-0.28584	0.574852	-0.03629	0.413477

Table S9. Loadings period B

Food groups derived from FFQ	Factor1	Factor2	Factor3	H² (Communality)
**Sugary sweets & Sugar beverages - B**	-0.0167	0.469626	0.020473	0.221247
**Non-Refined products and grains - B**	0.166666	-0.27216	0.230909	0.155169
**Processed foods - B**	0.152034	-0.00824	0.001323	0.023184
**Meat group - B**	0.078106	0.187968	0.073479	0.046832
**Beverages - B**	-0.02202	0.147968	0.278921	0.100176
**Fresh Juice - B**	0.336795	-0.00806	-0.07831	0.119628
**Alcohol - B**	-0.02693	0.006646	0.185017	0.035001
**Vegetables - B**	0.393775	0.280012	-0.01397	0.233661
**Fruits - B**	0.505897	-0.13944	0.062267	0.279253
**Fresh salad - B**	0.158741	-0.1916	0.215919	0.108531
**Boiled salad - B**	0.220957	-0.09782	0.171405	0.08777
**Eggs - B**	0.185287	-0.03227	0.185714	0.069862
**Legumes - B**	0.366106	-0.01647	-0.05689	0.137541
**Nuts and olives and oil - B**	0.48066	0.182059	0.180278	0.29668
**Fish and shellfish - B**	0.162358	-0.13339	0.095168	0.05321
**Plant-based dairy - B**	0.134417	-0.15365	0.041047	0.043362
**Dairy - B**	0.073309	0.16635	0.134437	0.05112
**Animal Fats - B**	0.109368	0.307615	0.113331	0.119432
**Plant Fats - B**	-0.02718	0.14701	0.028297	0.023151
**Sugar alternatives - B**	-0.01544	0.035504	0.334201	0.113189
**Water - B**	0.073028	-0.16882	0.007508	0.033888
**Ultra processed foods & ColdCut & Refined - B**	-0.05125	0.56134	-0.14075	0.33754

Table S10. Factors and adherence to MD.

Factors	aOR (95% CI)	p-value (aOR)	power (aOR)
**High adherence to MD**			
**Factor1 - A**	0.97 (0.7,1.33)	0.87	0.06

**Factor2 - A**	0.97 (0.58,1.6)	0.93	0.057
**Factor3 - A**	1.96 (1.31,3.02)	0.001**	1
**Factor1 - B**	1.05 (0.7,1.59)	0.8	0.09
**Factor2 - B**	0.88 (0.59,1.29)	0.53	0.28
**Factor3 - B**	1.43 (0.67,3.07)	0.34	0.979
**Medium adherence to MD**			
**Factor1 - A**	0.24	1.3 (0.84,2.06)	0.664
**Factor2 - A**	0.16	0.61 (0.29,1.19)	0.992
**Factor3 - A**	0.26	1.42 (0.76,2.65)	0.883
**Factor1 - B**	0.003**	2.91 (1.5,6.24)	1
**Factor2 - B**	0.001**	0.34 (0.17,0.64)	1
**Factor3 - B**	0.014*	4.94 (1.48,19.36)	1
**Low adherence to MD**			
**Factor1 - A**	0.082	1.24 (0.97,1.58)	0.855
**Factor2 - A**	0.28	0.8 (0.54,1.18)	0.858
**Factor3 - A**	0.63	1.08 (0.77,1.47)	0.195
**Factor1 - B**	0.27	1.22 (0.85,1.75)	0.804
**Factor2 - B**	0.29	0.84 (0.62,1.15)	0.646
**Factor3 - B**	0.008**	2.16 (1.23,3.85)	1

Table S11. GL associations with GDM in the high MD adherence group.

	**Period A - Before pregnancy**		**Period B - During pregnancy**	
**Food groups and their GL**	**aOR (95% CI)**	**p-value**	**aOR (95% CI)**	**p-value**
Sugary sweets & Sugar beverages	1.00 (0.99–1.01)	0.26	1.00 (0.99–1.00)	0.80
Non-Refined products and grains	1.00 (0.98–1.02)	0.87	1.00 (0.98–1.01)	0.61
Processed foods	1.00 (0.96–1.03)	0.86	1.00 (0.98–1.03)	0.55
Fresh Juice	0.97 (0.79–1.14)	0.79	1.00 (0.87–1.13)	0.97
Alcohol	1.12 (0.88–1.37)	0.27	1.30 (0.16–5.66)	0.75
Vegetables	0.95 (0.87–1.04)	0.37	0.98 (0.90–1.06)	0.69

Fruits	1.00 (0.97–1.02)	0.91	1.00 (0.98–1.02)	0.36
Fresh salad	1.72 (0.63–4.61)	0.28	1.28 (0.50–3.24)	0.59
Boiled salad	0.09 (0.01–0.73)	0.032*	0.11 (0.01–0.76)	0.039*
Legumes	0.71 (0.17–2.60)	0.63	0.81 (0.26–2.46)	0.72
Nuts and olives and oil	0.79 (0.40–1.48)	0.49	0.75 (0.37–1.42)	0.40
Plant-based dairy	0.29 (0.00–8.21)	0.51	1.73 (0.18–23.19)	0.64
Dairy	0.99 (0.88–1.07)	0.89	1.06 (0.94–1.20)	0.31
Animal Fats	5.45×10^{-11} (5.22×10^{-29}–5512.62)	0.20	9956.18 (1.23×10^{-8}–3.03×10^{14})	0.47
Sugar alternatives	0.98 (0.90–1.03)	0.65	0.64 (0.35–0.94)	0.076
Ultra processed & Cold cuts & Refined	0.99 (0.99–1.00)	0.72	0.99 (0.99–1.00)	0.53

*Food groups derived from FFQ

Table S12. GL associations with GDM in the medium MD adherence group.

	Period A - Before pregnancy		Period B - During pregnancy	
**Food groups and their GL**	**aOR (95% CI)**	**p-value**	**aOR (95% CI)**	**p-value**
Sugary sweets & Sugar beverages	0.99 (0.98,1)	0.82	0.98 (0.96,0.99)	0.05*
Non-Refined products and grains	1.01 (0.98,1.03)	0.41	1.02 (0.99,1.04)	0.13
Processed foods	1 (0.96,1.04)	0.68	0.99 (0.95,1.03)	0.93
Fresh Juice	1.14 (0.85,1.5)	0.34	0.89 (0.65,1.17)	0.44
Alcohol	1.28 (0.81,1.86)	0.2	0.3 (0,4.16)	0.56
Vegetables	1.05 (0.92,1.2)	0.4	1.03 (0.91,1.16)	0.6
Fruits	1 (0.96,1.03)	0.78	1.03 (1,1.07)	0.022*
Fresh salad	0.78 (0.14,3.87)	0.77	0.72 (0.17,2.88)	0.65
Boiled salad	0.25 (0.01,4.12)	0.37	12.66 (1.78,106.87)	0.014*
Legumes	1.25 (0.25,5.22)	0.76	9.51 (2.39,44.57)	0.002**
Nuts and olives and oil	1.23 (0.48,3.11)	0.65	1.72 (0.69,4.29)	0.23
Plant-based dairy	0.43 (0,26.37)	0.74	0.05 ($8.12*10^{-7}$,35.39)	0.48
Dairy	0.86 (0.71,1.01)	0.12	0.98 (0.83,1.1)	0.83
Animal Fats	$8.78*10^{-7}$ ($5.1*10^{-32}$,$1.4*10^{6}$)	0.55	0 ($3.49*10^{-28}$,$9.93*10^{14}$)	0.77
Sugar alternatives	0.93 (0.73,1.06)	0.39	0.97 (0.78,1.1)	0.72
Ultra processed & Cold cut & Refined	0.99 (0.99,1)	0.34	0.99 (0.99,0.99)	0.037*

*Food groups derived from FFQ

Table S13. GL associations with GDM in the low adherence group.

	Period A - Before pregnancy		Period B - During pregnancy	
**Food groups and their GL**	**aOR (95% CI)**	**p-value**	**aOR (95% CI)**	**p-value**
Sugary sweets & Sugar beverages	0.99 (0.99,1)	0.43	0.99 (0.98,1)	0.21

Non-Refined products and grains	1.02 (1.01,1.03)	p<0.001***	1.01 (1,1.02)	0.005**
Processed foods	1 (0.97,1.03)	0.61	0.97 (0.94,1)	0.17
Fresh Juice	0.91 (0.76,1.05)	0.29	0.98 (0.86,1.08)	0.72
Alcohol	1.02 (0.81,1.24)	0.82	1.53 (0.78,3.41)	0.22
Vegetables	0.99 (0.92,1.05)	0.83	0.96 (0.89,1.03)	0.3
Fruits	1 (0.97,1.02)	0.94	0.99 (0.97,1.01)	0.67
Fresh salad	0.96 (0.44,2)	0.93	1.51 (0.76,2.94)	0.23
Boiled salad	0.29 (0.04,1.56)	0.18	0.61 (0.11,2.74)	0.55
Legumes	1.43 (0.55,3.53)	0.44	1.27 (0.65,2.54)	0.47
Nuts and olives and oil	1.51 (0.89,2.54)	0.11	1.54 (0.9,2.64)	0.11
Plant-based dairy	0.18 (0,9.16)	0.48	0 ($1.59*10^{-7}$,1.32)	0.14
Dairy	1.02 (0.94,1.1)	0.58	1.03 (0.95,1.13)	0.39
Animal Fats	502.61 (0,$7.8*10^{8}$)	0.39	0 ($5.17*10^{-12}$,$1.03*10^{5}$)	0.53
Sugar alternatives	1.02 (0.96,1.07)	0.37	1.02 (0.95,1.09)	0.4
Ultra processed & Cold cut & Refined	0.99 (0.99,0.99)	0.027*	0.99 (0.99,1)	0.17

*Food groups derived from FFQ

Table S14. GL associations with GDM non-stratified (total population)

	**Period A - Before pregnancy**		**Period B - During pregnancy**	
**Food groups and their GL**	**aOR (95% CI)**	**p-value**	**aOR (95% CI)**	**p-value**
Sugary sweets & Sugar beverages	1 (0.99,1)	0.98	0.99 (0.99,1)	0.11
Non-Refined products and grains	1.01 (1,1.02)	0.005**	1.01 (1,1.02)	0.002**
Processed foods	1 (0.98,1.02)	0.78	0.99 (0.98,1.01)	0.74
Fresh Juice	0.97 (0.87,1.07)	0.63	0.98 (0.9,1.06)	0.71
Alcohol	1.08 (0.93,1.23)	0.25	1.45 (0.8,2.63)	0.19
Vegetables	0.99 (0.95,1.04)	0.94	0.98 (0.94,1.03)	0.56
Fruits	1 (0.98,1.01)	0.88	1 (0.99,1.01)	0.28
Fresh salad	0.96 (0.57,1.61)	0.91	1.15 (0.7,1.87)	0.56
Boiled salad	0.19 (0.05,0.63)	0.008**	0.69 (0.26,1.7)	0.44
Legumes	1.08 (0.56,2.02)	0.8	1.49 (0.9,2.49)	0.12
Nuts and olives and oil	1.22 (0.87,1.7)	0.23	1.23 (0.86,1.74)	0.24
Plant-based dairy	0.28 (0.01,2.66)	0.32	0.84 (0.11,4.5)	0.85
Dairy	0.99 (0.94,1.04)	0.83	1.02 (0.96,1.08)	0.46
Animal Fats	2.92 ($1.55*10^{-5}$,$1.24*10^{5}$)	0.85	0.2 ($9.79*10^{-8}$,$1.11*10^{5}$)	0.82
Sugar alternatives	0.99 (0.96,1.02)	0.91	0.99 (0.93,1.04)	0.83
Ultra processed & ColdCut & Refined	0.99 (0.99,0.99)	0.021*	0.99 (0.99,0.99)	0.037*

*Food groups derived from FFQ
